# The Prevalence and Severity of Computer Vision Syndrome Among Primary Care Health Workers in the Ministry of National Guard Health Affairs, Central Region, Saudi Arabia

**DOI:** 10.7759/cureus.74741

**Published:** 2024-11-29

**Authors:** Abdulaziz H Alenazi, Nada M Alshehri, Mohammed A Alshehri, Taghreed M Alhazmi

**Affiliations:** 1 Family Medicine and Primary Care Department, King Abdulaziz Medical City Riyadh, Riyadh, SAU

**Keywords:** computer vision syndrome (cvs), eye strain, healthcare professionals (hcws), health-care workers, primary health care workers

## Abstract

Background

Computer vision syndrome (CVS) is a common condition affecting individuals who spend prolonged periods using electronic devices. It is characterized by symptoms such as eye strain, dryness, headaches, and neck pain. This study aims to assess the prevalence of CVS symptoms among healthcare professionals, their awareness of the condition, and the effectiveness of various preventative practices.

Methodology

A cross-sectional survey was conducted among 233 healthcare professionals, including physicians, nurses, pharmacists, lab technicians, and non-medical staff. Participants were asked about their demographic characteristics, awareness of CVS, device usage patterns, and preventative practices. The prevalence of CVS symptoms and their association with demographic factors and preventative measures were analyzed using chi-square tests.

Results

The average age of participants was 37 years, with 94 male (40.3%) and 139 female (59.7%). A significant majority, 192 (82.2%), of the participants had not heard of CVS, and 174 (74.8%) were unsure if it was a permanent condition. The most commonly used devices were mobile phones (199, 85.5%), followed by computers/laptops/iPads (117, 50.2%). Common CVS symptoms included neck or shoulder pain (171, 73.4%), headaches (162, 69.5%), and eye dryness (149, 64.0%). Significant associations were found between wearing glasses or contact lenses and a higher prevalence of symptoms (p = 0.042). Taking breaks while using devices was significantly associated with fewer symptoms (p = 0.050). Although adjusting device brightness and using anti-glare filters approached significance, they were not statistically significant.

Conclusion

The study highlights a considerable lack of awareness about CVS among healthcare professionals and a high prevalence of symptoms. Preventative practices, particularly taking breaks, are crucial in reducing CVS symptoms. Educational initiatives to raise awareness and promote healthier visual habits are needed to mitigate the impact of CVS. Addressing these issues can enhance the well-being and productivity of healthcare professionals in digital environments.

## Introduction

Screens and computers have become an integral part of our daily lives as a result of the digital revolution, which made them an integral part of our lives. Nowadays, the use of mobile tools, computers, iPads, and smartphones is widespread and is being used by a vast number of people, including both children and adults, and has become one of the most common things, both at work and off work. Nevertheless, the use of digital devices may adversely affect the vision of users [1-4]. According to the American Optometric Association, the term computer vision syndrome (CVS) is defined by a complex of eye and vision problems related to near vision activities involving computer use. The most common symptoms associated with computer vision syndrome (CVS) are eyestrain, headaches, blurred vision, dry eyes, neck, and shoulder pain.

In addition, CVS incidence ranges from 64% to 90% in computer users. About 60 million people worldwide suffer from CVS, and about a million new CVS cases occur every year [5]. CVS can be reduced by maintaining an ideal distance from the screen, keeping the eyes at an ideal level above the screen's top, using anti-glare screens, and taking frequent breaks [6,7]. A study was conducted to identify the factors leading to CVS, which shows that the angle of gaze at the computer monitor was a major factor in developing CVS. It was found that gazing downwards at angles of 14 degrees or more was associated with less pain [8].

Moreover, a study that examined the prevalence and risk factors of CVS among computer office workers concluded that female gender, higher daily computer usage, not using a visual display terminal (VDT) filter, and wearing contact lenses were significantly associated with CVS symptoms [9]. Many studies have been conducted to determine the prevalence of computer vision syndrome (CVS). The majority of studies were conducted on office-based jobs. There are, however, only a few studies addressing CVS prevalence among healthcare professionals.

In Spain, a study was carried out on 622 healthcare workers, including physicians, surgeons, nurses, and nursing assistants. The prevalence of CVS was found to be 56.75%. When compared with other healthcare workers, nurses were the most affected group. A higher prevalence of CVS among nurses was associated with female sex, a seniority of 10-20 years, and the use of a video display terminal for over four hours daily. Female sex, morning shifts, and on-call were associated with an increased prevalence of CVS in the physicians and surgeons group [10].

Locally, a cross-sectional study that took place in 2021 was carried out to assess the prevalence of computer vision syndrome among all radiologists and radiology residents residing and practicing in Saudi Arabia. They found that 65.4% of the participants experienced symptoms of computer vision syndrome. Headache, dryness, burning, blurred vision, and increased sensitivity to light were the most reported symptoms [11]. A study conducted at Qassim University's College of Medicine with 634 students found that long-term computer use causes serious vision problems, especially for those who use them frequently [12].

One study that was carried out on the Saudi population between the period from July to December 2017 found that the prevalence of CVS was 77.6%. Eye burning was found to be the most common ocular symptom, while neck/shoulder pain was found to be the most prevalent extraocular symptom. A significant association was observed between the CVS symptoms and prolonged use of digital devices [13]. Until now, only one study has investigated CVS prevalence and whether there are differences among occupational groups when it comes to CVS in healthcare workers.

No local studies address the prevalence of CVS among healthcare workers. Thus, we aim to assess the prevalence and severity of CVS among healthcare workers in the department of family medicine and primary health care at the National Guard Hospital in Riyadh.

## Materials and methods

Study area/setting

This study was conducted at King Abdulaziz Medical City in Riyadh (KAMC-R), specifically within the primary care centers.

Study subjects

The study subjects were primary care health workers at King Abdulaziz Medical City, Riyadh region (KAMC). The inclusion criteria comprised all primary care health workers within the primary care setting at KAMC. The exclusion criteria were limited to pregnant workers who were not included in the study.

Study design

This research employed a cross-sectional study design. This design was chosen to assess the prevalence and characteristics of computer vision syndrome (CVS) among primary care health workers.

Sample size

The total number of healthcare workers (HCWs) at the study site was 1120, as advised by the CM department management. The prevalence of CVS was considered based on a previous study conducted in Riyadh in 2019. Using a 95% confidence interval, the required sample size was calculated to be 287 participants.

Sampling technique

Convenience sampling was utilized to select participants from the target population. This technique was chosen for its practicality and ease of implementation in a healthcare setting.

Data collection methods, instruments used, and measurements

Data collection was carried out using a handed questionnaire designed to gather relevant information. The questionnaire included sections on demographics, screen exposure details, symptoms of CVS, and preventive measures (Appendix 1). The source of the questionnaire was another study, but it was modified to suit the specific needs of this research [14,15]. A permission from the original publishers to modify and adapt the questionnaire for the current study was obtained. Three experts in the field validated the structure of the questionnaire to ensure its appropriateness and reliability.

Data management and analysis plan

The collected data was initially entered into Microsoft Excel (Microsoft Corporation, Redmond, Washington, United States) and subsequently exported to IBM SPSS Statistics for Windows, Version 21 (Released 2012; IBM Corp., Armonk, New York, United States) for analysis. Numerical data were described using means and standard deviations, while categorical data were described using frequencies and percentages. The chi-square test was employed to compare categorical data, and the T-test was used to assess the relationship between numerical and categorical variables. A p-value of less than 0.05 was considered indicative of statistical significance in this study.

## Results

The demographic characteristics of the participants are summarized in Table 1. The average age of the participants was 37 years, with a standard deviation of 9.02 years. Among the participants, 40.3% were male and 59.7% were female. Regarding marital status, 34.3% were single, 60.2% were married, 5.1% were divorced, and 0.4% were widowed. In terms of occupation, 35.5% of the participants were physicians, 40.4% were nurses, 14.8% were pharmacists, and 9.3% were lab technicians. For non-medical occupations, 13.2% were administrative assistants, 22.6% were in health information management, 35.8% were in inpatient services, 3.8% were medical technologists, and 24.5% were in administration. When asked about computer vision syndrome (CVS), 82.2% of the participants had not heard of it, while 17.8% had. Regarding whether CVS is a permanent condition, 19.7% of the participants believed it is not permanent, 5.6% thought it is permanent, and 74.8% did not know (Table 1).

**Table 1:** Demographic factors of the participants

		Count	Column N %
Age	Mean (SD)	37.0 (9.02)	
Gender	Male	94	40.3%
	Female	139	59.7%
Marital status	Single	81	34.3%
	Married	142	60.2%
	Divorced	12	5.1%
	Widow	1	0.4%
Medical occupation	Physician	65	35.5%
	Nursing	74	40.4%
	Pharmacist	27	14.8%
	Lab technician	17	9.3%
Non-medical occupation	Admin assistant	7	13.2%
	Health information management	12	22.6%
	Patient services	19	35.8%
	Medical technologist	2	3.8%
	Administration	13	24.5%
Computer vision syndrome: Have you heard of it?	No	194	82.2%
	Yes	42	17.8%
Do you think computer vision syndrome is a permanent condition?	No	46	19.7%
	Yes	13	5.6%
	I don't know	175	74.8%

**Figure 1:**
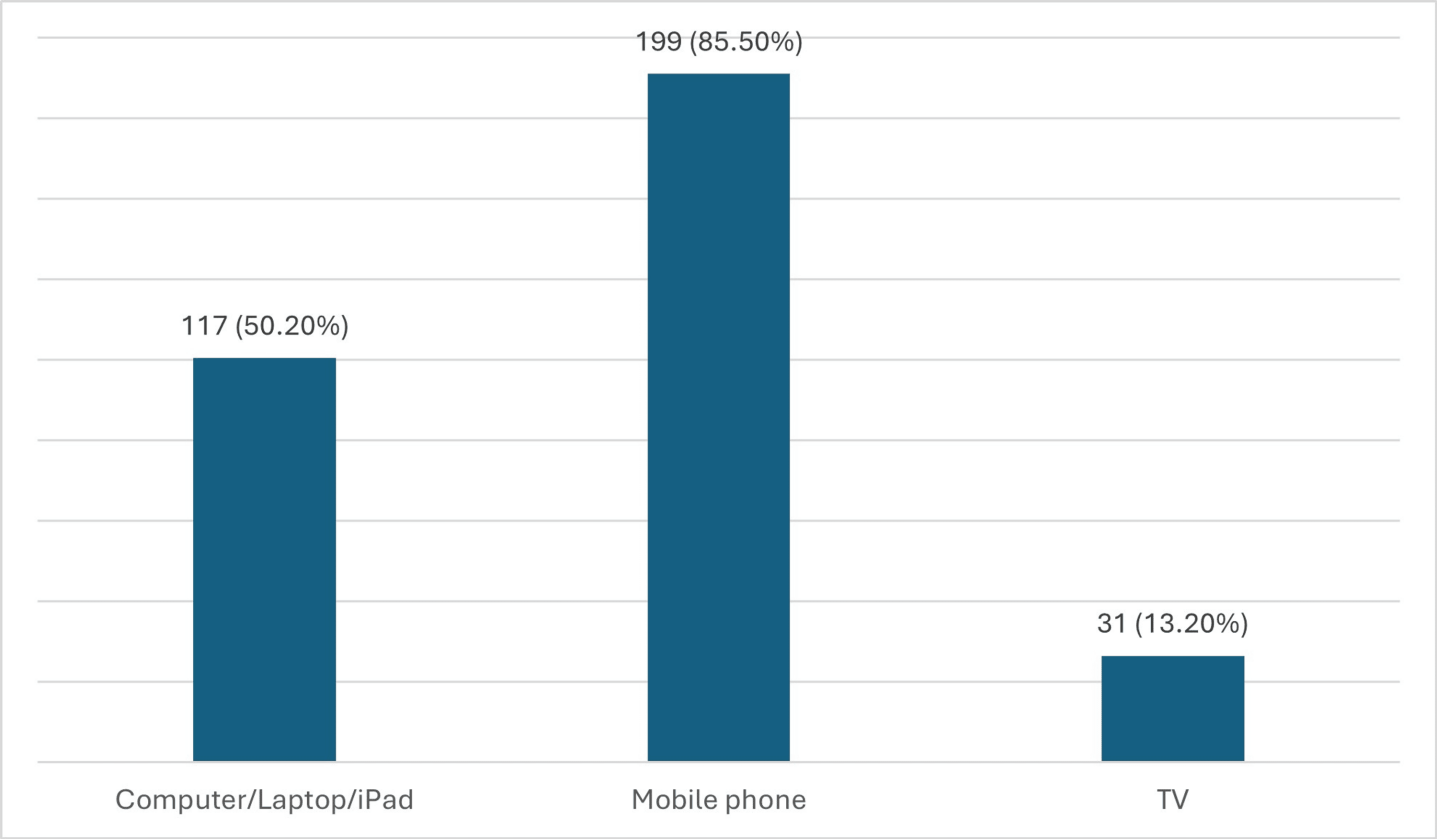
Which type of device do you spend the most time on?

Participants reported the types of devices they spent the most time on 50.2% used computers, laptops, or iPads, 85.5% used mobile phones, and 13.2% watched TV (Figure 1).

Regarding preventative practices against computer vision syndrome (CVS), 74.6% of participants had not been diagnosed with any refractive error, and 64.3% did not wear glasses or contact lenses. Adjusting device brightness based on surrounding lighting was practiced by 69.8% of participants, and 76.2% took breaks while using devices. Additionally, 66.8% kept the screen at their face level, and 64.3% sat with the top of the screen at eye level. However, 52.3% did not keep the device screen more than 50 cm away, and 72.1% did not use anti-glare filters. When asked if they believed CVS could be prevented, 54.5% said yes, 5.1% said no, and 40.4% did not know. Regarding the improvement of CVS symptoms with healthier visual habits, 43.8% agreed, 3.4% disagreed, and 52.8% did not know. Finally, when asked about their willingness to decrease screen hours to prevent CVS, 48.5% agreed, 4.7% strongly agreed, 35.3% were neutral, 6.8% disagreed, and 4.7% strongly disagreed (Table 2).

**Table 2:** Preventative practice against CVS CVS: computer vision syndrome

		Count	Column N %
Have you been previously diagnosed with any refractive error?	No	173	74.6%
	Yes	59	25.4%
Are you wearing glasses or contact lenses?	No	151	64.3%
	Yes	84	35.7%
Do you adjust the device brightness based on the surrounding lighting?	No	71	30.2%
	Yes	164	69.8%
Do you take breaks while using the device?	No	56	23.8%
	Yes	179	76.2%
Do you have the screen on the level of your face?	No	78	33.2%
	Yes	157	66.8%
Do you sit while the top of the screen is on your eye level?	No	84	35.7%
	Yes	151	64.3%
Do you have the device screen more than 50 cm away?	No	123	52.3%
	Yes	112	47.7%
Do you use anti-glare filters?	No	168	72.1%
	Yes	65	27.9%
Do you think computer vision syndrome can be prevented?	No	12	5.1%
	Yes	128	54.5%
	I don't know	95	40.4%
Do you think symptoms of CVS improve with healthier visual habits?	No	8	3.4%
	Yes	103	43.8%
	I don't know	124	52.8%
I’m willing to decrease my screen hours to guard against CVS	Strongly disagree	11	4.7%
	Disagree	16	6.8%
	Neutral	83	35.3%
	Agree	114	48.5%
	Strongly agree	11	4.7%

**Figure 2:**
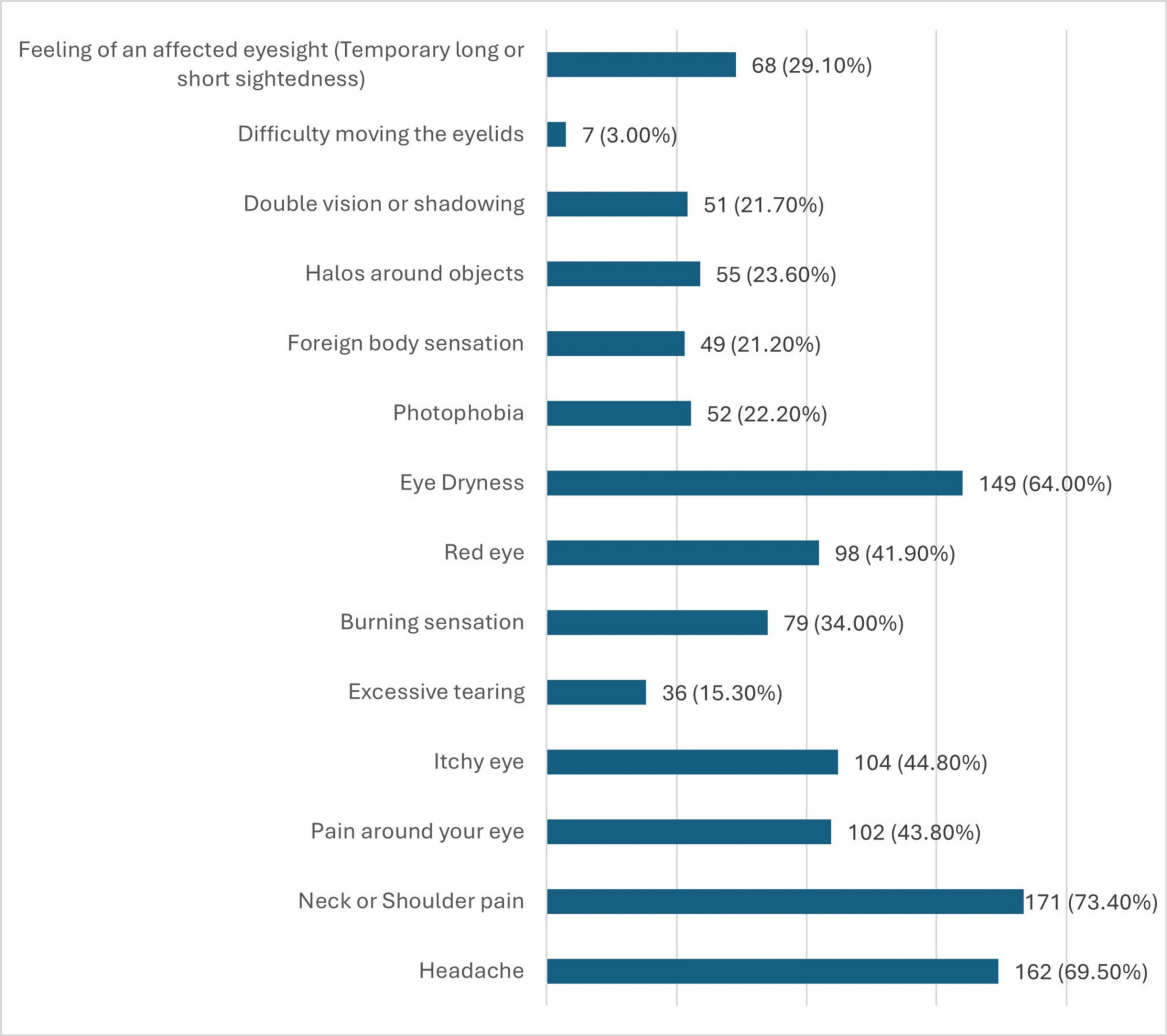
In the past three months, did you experience any of the following symptoms after the use of electronic devices?

In the past three months, participants reported experiencing various symptoms after using electronic devices. The most prevalent symptoms were neck or shoulder pain (73.4%) and headache (69.5%). Eye dryness was also highly reported, affecting 64.0% of participants. Pain around the eyes was experienced by 43.8%, and itchy eyes by 44.8%. Red eyes were noted by 41.9% of participants, followed by a burning sensation (34.0%) and temporary changes in vision, such as a feeling of affected eyesight (29.1%). Other symptoms included halos around objects (23.6%), photophobia (22.2%), a foreign body sensation (21.2%), and double vision or shadowing (21.7%). Less commonly reported symptoms, such as excessive tearing (15.3%) and difficulty moving the eyelids (3.0%), were collectively noted by a smaller portion of participants (Figure 2).

The relation between the prevalence of eye symptoms and demographic factors was analyzed. There was no significant association between gender and the number of eye symptoms (p = 0.066), with both males and females experiencing symptoms similarly across different ranges. Marital status also showed no significant difference in symptom prevalence (p = 0.900), with single, married, and divorced participants reporting similar distributions of symptoms. We found no significant differences in symptom prevalence (p = 0.910) among medical occupations, with physicians, nurses, pharmacists, and lab technicians experiencing a comparable range of symptoms. Non-medical occupations also did not show significant differences (p = 0.383). Interestingly, wearing glasses or contact lenses was significantly associated with a higher prevalence of symptoms (p = 0.042). Participants who wore corrective lenses reported more symptoms compared to those who did not (Table 3).

**Table 3:** The relation between prevalence of eye symptoms and demographic factors Analyzed using the chi-square test. A p-value of less than 0.05 was considered indicative of statistical significance (*).

		Symptoms						
		0-2		3-7		> 7		P-value
		Count	Row N %	Count	Row N %	Count	Row N %	
Gender	Male	36	38.3%	41	43.6%	17	18.1%	0.066
	Female	37	26.6%	82	59.0%	20	14.4%	
Marital status	Single	25	30.9%	45	55.6%	11	13.6%	0.900
	Married	47	33.1%	70	49.3%	25	17.6%	
	Divorced	3	25.0%	7	58.3%	2	16.7%	
	Widow	0	0.0%	1	100.0%	0	0.0%	
Medical occupation	Physician	22	33.8%	34	52.3%	9	13.8%	0.910
	Nursing	23	31.1%	36	48.6%	15	20.3%	
	Pharmacist	7	25.9%	14	51.9%	6	22.2%	
	Lab technician	4	23.5%	10	58.8%	3	17.6%	
Non-medical occupation	Admin assistant	2	28.6%	4	57.1%	1	14.3%	0.383
	Health information management	4	33.3%	8	66.7%	0	0.0%	
	Patient services	5	26.3%	11	57.9%	3	15.8%	
	Medical technologist	0	0.0%	2	100.0%	0	0.0%	
	Administration	8	61.5%	4	30.8%	1	7.7%	
Computer vision syndrome: have you heard of it?	No	61	31.4%	103	53.1%	30	15.5%	0.935
	Yes	14	33.3%	21	50.0%	7	16.7%	
Computer/Laptop/iPad	Yes	26	22.0%	70	59.3%	22	18.6%	0.352
Mobile phone	Yes	62	30.8%	110	54.7%	29	14.4%	
TV	Yes	9	29.0%	17	54.8%	5	16.1%	
Have you been previously diagnosed with any refractive error?	No	59	34.1%	90	52.0%	24	13.9%	0.133
	Yes	13	22.0%	33	55.9%	13	22.0%	
Are you wearing glasses or contact lenses?	No	52	34.4%	80	53.0%	19	12.6%	0.042*
	Yes	21	25.0%	44	52.4%	19	22.6%	

The relationship between the prevalence of eye symptoms and preventative practices against computer vision syndrome (CVS) was analyzed. Adjusting device brightness based on surrounding lighting did not show a significant association with symptom prevalence (p = 0.153). However, taking breaks while using devices was significantly associated with a lower prevalence of symptoms (p = 0.050), indicating that participants who took breaks reported fewer symptoms. The position of the screen relative to the participant's face and eye level did not show significant associations with symptom prevalence (p = 0.804 and p = 0.201, respectively). The distance of the device screen from the participant also did not have a significant impact on symptom prevalence (p = 0.321). Using anti-glare filters approached significance (p = 0.054), suggesting a potential trend where those who used anti-glare filters reported fewer symptoms compared to those who did not (Table 4).

**Table 4:** The relation of prevalence of eye symptoms and preventative practice against CVS Analyzed using the chi-square test. A p-value of less than 0.05 was considered indicative of statistical significance (*). CVS: computer vision syndrome

		Symptoms						
		0-2		3-7		> 7		P-value
		Count	Row N %	Count	Row N %	Count	Row N %	
Do you adjust the device brightness based on the surrounding lighting?	No	21	29.6%	43	60.6%	7	9.9%	0.153
	Yes	52	31.7%	81	49.4%	31	18.9%	
Do you take breaks while using the device?	No	10	17.9%	35	62.5%	11	19.6%	0.050*
	Yes	63	35.2%	89	49.7%	27	15.1%	
Do you have the screen on the level of your face?	No	24	30.8%	43	55.1%	11	14.1%	0.804
	Yes	49	31.2%	81	51.6%	27	17.2%	
Do you sit while the top of the screen is on your eye level?	No	20	23.8%	49	58.3%	15	17.9%	0.201
	Yes	53	35.1%	75	49.7%	23	15.2%	
Do you have the device screen more than 5No cm away?	No	33	26.8%	68	55.3%	22	17.9%	0.321
	Yes	40	35.7%	56	50.0%	16	14.3%	
Do you use anti-glare filters?	No	45	26.8%	94	56.0%	29	17.3%	0.054
	Yes	28	43.1%	29	44.6%	8	12.3%	

## Discussion

The study provides a comprehensive analysis of the prevalence of computer vision syndrome (CVS) symptoms and their association with demographic factors and preventative practices among healthcare professionals. The findings highlight the widespread lack of awareness about CVS and underscore the importance of adopting healthier visual habits to mitigate its effects.

The majority of participants (82.2%) had not heard of CVS, reflecting a significant gap in awareness. This is higher than reported in a previous study conducted by Rukundo P, who reported that 52.3% reported not hearing about CVS [16]. However, the low level of awareness was also reported in a previous Saudi study [17]. This lack of knowledge is concerning given the high prevalence of CVS symptoms reported in the study. The finding that 74.8% of participants were unsure if CVS is a permanent condition further emphasizes the need for educational initiatives to inform healthcare workers about CVS and its potential long-term effects.

Participants reported high usage of electronic devices, with 85.5% using mobile phones, 50.2% using computers, laptops, or iPads, and 13.2% watching TV. This pattern is consistent with global trends showing increasing reliance on digital devices in both personal and professional settings [18]. The high use of mobile phones is particularly noteworthy, as it suggests that interventions to reduce CVS should consider mobile usage habits specifically.

The study identified neck or shoulder pain (73.4%) and headache (69.5%) as the most prevalent symptoms, followed by eye dryness (64.0%), itchy eyes (44.8%), and red eyes (41.9%). These findings align with previous research indicating that prolonged use of digital devices can lead to significant discomfort and visual disturbances [19,20]. This aligns with findings from other studies, which identified headaches as the most frequent symptom associated with computer use [9,21-24]. Conversely, a study among bank workers in Pakistan reported body fatigue as the most common issue due to prolonged computer use, with half of the participants using computers for more than eight hours daily, likely due to extended periods of sitting during work hours [25,26]. In our study, the most commonly reported ocular symptoms were eye burning and itching, with prevalence rates of 68.3% and 63.6%, respectively. Severe eye itching was reported by 22.2% of participants, while severe eye burning was noted by 19.2%. These findings are consistent with the study in Pakistan, which found a 77.2% prevalence of eye burning [26]. The high prevalence of eye dryness is particularly concerning, as it can lead to more severe ocular conditions if not managed properly [27].

The analysis of preventative practices revealed several key insights. A significant proportion of participants (69.8%) adjusted device brightness based on surrounding lighting, and 76.2% took breaks while using devices. These practices are recommended to reduce CVS symptoms, as adjusting brightness can reduce glare, and taking breaks can prevent eye strain [28]. However, the study found that 52.3% of participants did not keep the device screen more than 50 cm away, and 72.1% did not use anti-glare filters. These practices are important for reducing the risk of CVS, as proper screen distance and the use of anti-glare filters can significantly reduce visual discomfort [28].

Gender, marital status, and occupation did not show significant associations with the prevalence of CVS symptoms. This suggests that CVS is a widespread issue affecting various demographic groups similarly, which underscores the universal need for preventative measures across different populations. In contrast, a study conducted in Sri Lanka revealed a significant predominance of CVS among females over a one-year period [9]. Another study from the United Arab Emirates, focusing on university students, indicated gender differences in specific CVS symptoms, with headaches being more frequently reported by female students [29]. A study involving university administrative staff in Ghana found a higher prevalence of CVS in males; however, the author suggested that this could be attributed to the unequal gender distribution within their study sample [30].

Interestingly, the study found a significant association between wearing glasses or contact lenses and a higher prevalence of symptoms (p = 0.042). This may be due to the fact that individuals with refractive errors already have a predisposition to visual discomfort, which can be exacerbated by prolonged screen use [19]. It highlights the need for these individuals to be particularly vigilant about adopting healthy visual habits.

Among the preventative practices analyzed, taking breaks while using devices was significantly associated with a lower prevalence of symptoms (p = 0.050). This finding supports the recommendation that regular breaks can help mitigate the effects of prolonged screen use by allowing the eyes to rest and recover [31]. The 20-20-20 rule, which suggests looking at something 20 feet away for 20 seconds every 20 minutes, is a practical strategy that can be easily implemented in daily routines [31].

Adjusting device brightness based on surrounding lighting approached some significance (p = 0.153), indicating a potential trend where those who adjusted brightness reported fewer symptoms. This practice can reduce the contrast between the screen and the surrounding environment, thereby reducing eye strain [32,33]. Using anti-glare filters also showed a trend towards significance (p = 0.054), suggesting that they may help reduce CVS symptoms. Anti-glare filters can minimize reflections and glare, which are common sources of visual discomfort when using screens [19,34].

The findings of this study have several implications for practice. First, there is a clear need for educational initiatives to raise awareness about CVS and its prevention. Healthcare organizations should consider implementing training programs to educate staff about the symptoms of CVS and effective strategies to prevent it. Second, promoting the adoption of healthy visual habits is crucial. Encouraging practices such as taking regular breaks, adjusting screen brightness, and using anti-glare filters can help reduce the prevalence of CVS symptoms. Healthcare facilities can also consider ergonomic interventions, such as providing adjustable workstations and promoting the use of appropriate lighting. Third, given the high use of mobile phones, specific interventions targeting mobile device usage should be developed. This could include guidelines on optimal viewing distances and breaks, as well as the promotion of applications that remind users to take breaks. Finally, individuals who wear glasses or contact lenses should be particularly vigilant about adopting preventative practices. Eye care professionals should provide tailored advice to these individuals to help them manage their visual comfort effectively.

This study has some limitations that should be considered. The cross-sectional design does not allow for causal inferences to be made. Future longitudinal studies are needed to explore the causal relationships between device use, preventative practices, and CVS symptoms. Additionally, the study relied on self-reported data, which may be subject to recall bias. Further research should also explore the effectiveness of specific interventions in reducing CVS symptoms. For example, randomized controlled trials could be conducted to evaluate the impact of anti-glare filters or specific break schedules on visual comfort and productivity.

## Conclusions

In conclusion, this study highlights the high prevalence of CVS symptoms among healthcare professionals and the importance of adopting preventative practices. Raising awareness and promoting healthy visual habits are essential steps in mitigating the impact of CVS and ensuring the well-being of individuals in digital environments. By addressing these issues, healthcare organizations can help reduce the burden of CVS and improve the quality of life for their staff.

## Appendices

Appendix 1

The questionnaire used in this study included sections on demographics, screen exposure details, symptoms of CVS, and preventive measures [35].
